# Treatment Failure and Overall Survival in Patients with Sinonasal Squamous Cell Carcinoma (SNSCC): A Systematic Review and Meta-Analysis

**DOI:** 10.3390/cancers18060948

**Published:** 2026-03-13

**Authors:** Urszula Kacorzyk, Aleksandra Krzywon, Magdalena Szymala-Cortez, Alexander Jorge Cortez, Krzysztof Składowski, Tomasz Rutkowski

**Affiliations:** 1 Radiation and Clinical Oncology Department, Maria Sklodowska-Curie National Research Institute of Oncology, Gliwice Branch, Wybrzeże Armii Krajowej 15, 44-102 Gliwice, Poland; krzysztof.skladowski@gliwice.nio.gov.pl; 2 Department of Biostatistics and Bioinformatics, Maria Sklodowska-Curie National Research Institute of Oncology, Gliwice Branch, Wybrzeże Armii Krajowej 15, 44-102 Gliwice, Poland; aleksandra.krzywon@gliwice.nio.gov.pl (A.K.); alexander.cortez@gliwice.nio.gov.pl (A.J.C.); 3 Radiotherapy Department, Maria Sklodowska-Curie National Research Institute of Oncology, Gliwice Branch, Wybrzeże Armii Krajowej 15, 44-102 Gliwice, Poland; magdalena.szymala-cortez@gliwice.nio.gov.pl; 4 Department of Science and Clinical Research Coordination, Maria Sklodowska-Curie National Research Institute of Oncology, Gliwice Branch, Wybrzeże Armii Krajowej 15, 44-102 Gliwice, Poland; tomasz.rutkowski@gliwice.nio.gov.pl

**Keywords:** squamous cell carcinoma of sinonasal region (SNSCC), sinonasal carcinoma, malignant neoplasms of the sinonasal tract, sinonasal malignancy, review, meta-analysis

## Abstract

**Objective:** Prognosis in SNSCC remains poor mainly for local failure. Due to the rarity of this disease, and heterogeneity as to site and stage, most of the existing studies are based on the experience of individual centers; as such, data from meta-analysis may be of special value. In this meta-analysis, we evaluated patterns of treatment failure—local recurrence (LR), nodal recurrence (NR), and distant metastasis (DM)—and their relationship with overall survival (OS). **Data Source:** A comprehensive search was performed in PubMed/MEDLINE, Web of Science, and Scopus for studies definitely involving treated patients with SNSCC. **Review Methods:** This study was conducted in accordance with the PRISMA 2000 Statement for Reporting Systematic Reviews Proportional meta-analysis to obtain pooled effect estimates, expressed as OS, local control (LC), distant metastasis-free survival (DMFS), disease-specific survival (DSS), LR rate, nodal recurrence (NR) rate and distant metastases (DM) rate. Rates of LR, NR and DM were analyzed and correlated with 5-year OS. **Results:** A total of 749 patients in 13 articles were identified. Multimodal treatment was administered in 588 cases (78%), including induction chemotherapy in 300 cases (40%), while 356 patients (47.5%) were treated with surgery followed by adjuvant radiotherapy (RT) or chemoradiotherapy (CHRT). The remaining 393 patients (52.5%) were treated without surgery. The pooled 2-, 3-, 5- and 10-year OS rates were 74%, 51.7%, 50.2% and 46.3%, respectively. The pooled 5-year local control (5-LC) rate was 57%, the pooled LR rate was 27.2% and the pooled NR rate was 11.6%. There was no statistically significant correlation between LR, NR, or DM and 5-year OS. **Conclusions:** SNSCC remains an aggressive disease with approximately 50% 5-year OS and LR as the main reason of treatment failure, with a rate of 27%. This confirms the necessity of better local control strategies.

## Simple Summary

Sinonasal squamous cell carcinoma (SNSCC) is the most common malignant tumor of the sinonasal region and remains associated with poor survival. In this meta-analysis, we evaluated patterns of treatment failure—local recurrence (LR), nodal recurrence (NR), and distant metastasis (DM)—and their relationship with overall survival (OS). Across 13 studies including 749 patients, LR was the predominant mode of failure (27%), whereas NR and DM were less frequent. No significant correlations between recurrence rates and OS were observed, likely reflecting heterogeneity and limited reporting across studies. These findings confirm the dominant role of local failure in SNSCC and highlight the need for improved local control strategies.

## 1. Introduction

Sinonasal squamous cell carcinoma (SNSCC) is the most common histopathological subtype of malignant tumors of the sinonasal region.

Despite advances in treatment techniques and improvements in surgical and radiotherapy procedures, the prognosis for this patient group remains poor mainly for local failure [1,2,3,4]. The rarity of SNSCC and its heterogeneity as to primary locations and varying clinical stages mean that most of the existing studies are based on the experience of individual centers with a small group size. Because of this, the results of SNSCC treatment have also been summarized in few systemic reviews and meta-analyses [2,5,6], population-based comparisons [3,7,8], or comprehensive comparative analyses [9]. All of them, however, refer to data from before 2020, concentrate on a selected site of the sinonasal region [5,6] or assess mixed histopathological subtypes [3,7,8,9].

In this meta-analysis, we evaluated patterns of treatment failure—local recurrence (LR), nodal recurrence (NR), and distant metastasis (DM)—and their relationship with overall survival (OS), collating all patients with SNSCC of all sites in the sinonasal region.

## 2. Material and Methods

This study was conducted in accordance with the PRISMA Statement for Reporting Systematic Reviews and Meta-Analyses of Studies that Evaluate Health Care Interventions, issued in 2009 [10]. The systematic review was not registered. This systematic review and meta-analysis investigated overall survival (OS) at 2, 3, 5, and 10 years, as well as 5-year local control (LC), local recurrence (LR) rate, nodal recurrence (NR) rate, and 5-year distant metastasis-free survival (DMFS), distant metastasis (DM) rate, and 5-year disease-specific survival (DSS) in radically treated SNSCC patients.

### 2.1. Literature Search Strategy

A systematic search was conducted in MEDLINE (PubMed), Scopus and Web of Science databases for studies published up to 30 November, 2025. The search included the following core terms: ‘Cancer of maxilla and sinonasal region’, ‘Sinonasal malignancy’, ‘Neoplasms of the maxillo-ethmoid massif’, ‘Maxillary sinus squamous cell carcinoma’, ‘Cancer of nasal cavity and paranasal sinuses’, ‘Sinonasal cancer’, ‘Paranasal sinuses malignancies’, ‘Nasal and paranasal sinus carcinoma’, ‘Malignant tumors of the nasal cavity, ethmoid and sphenoid sinuses’, ‘Malignant neoplasms of the sinonasal tract’, ‘paranasal sinus neoplasms, ‘maxillary sinus neoplasms’, ‘ethmoid sinus neoplasms’, ‘sphenoid sinus neoplasms’, ‘frontal sinus neoplasms’, ‘surgery’, ‘radiotherapy’, ‘radical treatment’, and ‘definitive treatment’. Detailed search strategies are presented in Supplementary Materials S1 (Tables S1–S4).

### 2.2. Eligibility Criteria

Studies were included if they met the following criteria: (1) patients aged ≥18 years (single cases of children were allowed), (2) patients diagnosed with SNSCC, (3) patients who underwent radical treatment (surgery or radiotherapy), (4) studies with a sample size of ≥15 patients, (5) studies reporting at least one of the following endpoints (OS, LC, nodal control (NC), DM, DSS, LR rate, NR rate, DM rate). Studies were excluded if they (1) included only pediatric patients (<18 years), (2) were not published in English, (3) were reviews, meta-analyses, case reports, or conference abstracts, (4) were published before 1 January 2000, (5) used data from the Surveillance, Epidemiology, and End Results (SEER) database or other national databases, or (6) were multi-center studies containing data from different countries.

### 2.3. Data Selection and Extraction

Two independent investigators (A.J.C. and A.K.) conducted the literature search based on both restrictive (quotation-based) and liberal approaches (without quotation marks) (Supplementary File S1 (Tables S1–S4)). Articles were collected in the data management software EndNote X7 (Clarivate Analytics (US) LLC, Philadelphia, PA, USA) (A.J.C.) and Mendeley Reference Manager v. 2.77.0 (Elsevier, Amsterdam, The Netherlands) (A.K.). Publication databases were combined and duplicates were removed using R environment (R Foundation for Statistical Computing, Vienna, Austria, http://www.r-project.org). The titles and abstracts of the remaining studies were independently screened by U.K. and M.S.-C. based on the inclusion and exclusion criteria using the retools package (v. 0.4.1) [11]. Full texts of eligible studies were further screened by U.K. and M.S.-C. Disagreements were adjudicated by T.R. Outcome data included OS, LC, NC, DM, and DSS at 2, 3, 5, and 10 years, as well as LR rate, NR rate and DM rate.

### 2.4. Risk of Bias

The risk of bias was independently assessed by two authors (U.K. and M.Sz.-C) using Cochrane’s Risk of Bias in Non-Randomized Studies of Interventions (ROBINS-I) tool [12]. Each bias domain in non-randomized studies was classified as “low”, “moderate”, “serious,” or “critical” risk. The following seven domains were evaluated: “confounding”, “selection of participants”, “classification of interventions”, “deviations from intended interventions”, “missing data”, “measurement of outcomes”, and “selection of reported result”. The risk of bias was visualized with the Risk-of-bias VISualization (robvis) tool [13].

### 2.5. Statistical Analysis

Proportional meta-analysis was used to obtain pooled effect estimates, expressed as OS, LC, DMFS, DSS, LR rate, NR rate and DM rate. The number of events (failures) and the number of surviving patients at 2, 3, 5, and 10 years were calculated as percentages multiplied by sample size. Forest plots with a 95% confidence interval (CI) were generated and visualized. The random-effects model with double arcsine transformation (Freeman–Tukey transformation) was used. The level of heterogeneity across studies was evaluated with the Cochrane Q test and I2 statistics. Heterogeneity was considered low if I2 < 30%, moderate if I2 30–75%, and high if I2 > 75% [14]. Significant heterogeneity was indicated by *p* < 0.05 in the Cochrane Q test. Spearman correlation coefficients were classified as follows: 0.0 ≤ |r| < 0.1 (negligible correlation), 0.1 ≤ |r| ≤ 0.39 (weak correlation), 0.4 ≤ |r| ≤ 0.69 (moderate correlation), 0.7 ≤ |r| ≤ 0.89 (strong correlation), and 0.9 ≤ r ≤ 1 (very strong correlation). All computational analyses were performed in the R environment for statistical computing (version 4.1.1 released on 10 August 2021; R Foundation for Statistical Computing, Vienna, Austria, http://www.r-project.org) (Accessed on 9 March 2026).

## 3. Results

### 3.1. Study Selection and Characteristics

**Figure 1:**
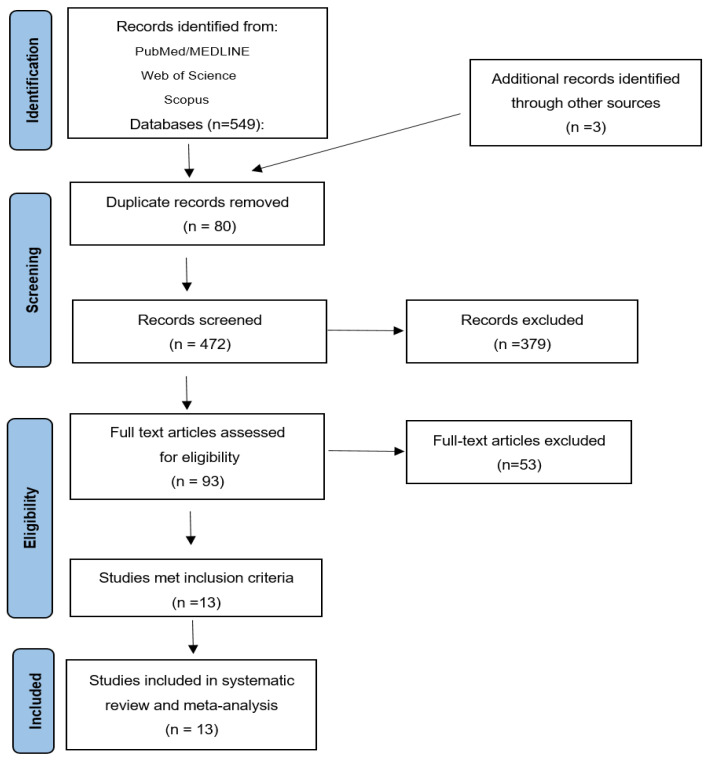
Flow diagram of the study selection.

A search strategy yielded 549 articles from PubMed/MEDLINE, Web of Science and Scopus, with an additional 3 articles identified through other sources. After excluding 80 duplicate records, 472 articles were screened based on titles and abstracts. Ninety-three articles were fully reviewed. A total of 13 studies fulfilled the eligibility criteria and were included in the final meta-analysis (Figure 1). These selected studies comprised a total of 749 patients, with sample sizes ranging from 18 to 123 patients per article [15,16,17,18,19,20,21,22,23,24,25,26,27]. The studies were conducted in Europe [17,20,21,24], North America [15,23], or Asia [16,18,19,25,26,27]. All studies were published between 2009 and 2023. Patients were diagnosed between 1988 and 2017, with a median follow-up of 36 months [IQR: 32.5–43] [15,16,17,18,19,20,21,22,23,24,25,26,27]. The median age of patients was 60 years [IQR: 59–60]. Key information, including authors, year of publication, country, sample size, patient characteristics, details of histopathological subtype, median follow-up, and outcomes, is presented in Table 1.

Among a subgroup of 646 patients with sufficient data, the distribution across T stages was as follows: T1—2%, T2—5.4%, T3—14%, and T4—78.6%. Nodal involvement (N+) was reported in 22.8% of patients. All patients in the cohort received radical treatment.

Multimodal treatment (at least two methods of surgery, RT, CHRT, iCH) was administered in 588 cases (78%), while single-modality treatment was used in 161 cases (22%). Across the entire cohort, 356 patients (47.5%) were treated with surgery followed by adjuvant RT or CHRT. CHRT or RT instead of surgery was used in 393 patients (52.5%). Particle beam radiation therapy (PT) or carbon ion therapy (CIT) were used in 65 (9%) and 21(3%) patients, respectively. Induction chemotherapy was administered in 300 cases (40%). Of these, 156 patients (52%) received iCH prior to surgery, and 144 patients (48%) received it prior to RT or CHRT (Table 1).

### 3.2. Overall Survival

**Figure 2:**
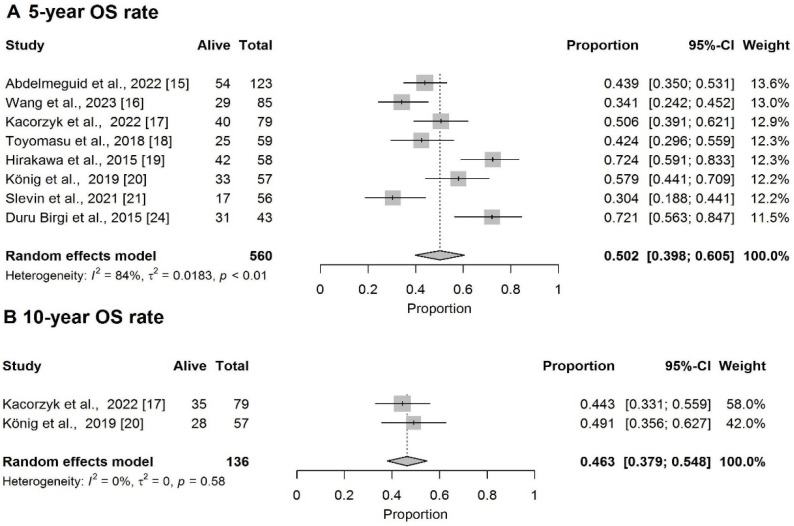
Forest plot of (**A**) 5-year OS rate [15,16,17,18,19,20,21,24] and (**B**) 10-year OS rate [17,20].

The pooled 2-year and 3-year OS rates were 74% (95%CI: 63.5–83.0%) and 51.7% (95%CI: 40.8–62.4%), respectively (Supplementary Materials S1, Figures S1 and S2). The pooled 5-year and 10-year OS rates were 50.2% (95%CI: 39.8–60.5%) and 46.3% (95%CI: 37.9–54.8%) (Figure 2A,B).

### 3.3. Local Control

**Figure 3:**
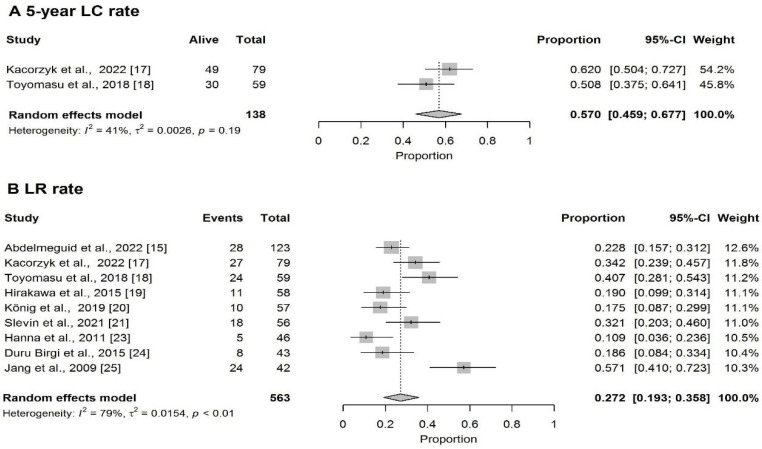
Forest plot of (**A**) 5-year LC rate [17,18] and (**B**) LR rate [15,17,18,19,20,21,23,24,25].

The pooled 5-year LC rate was 57% (95%CI: 45.9–67.7%) (Figure 3A) and the pooled LR rate was 27.2% (95%CI: 19.3–35.8%) (Figure 3B).

### 3.4. Nodal Control

**Figure 4:**
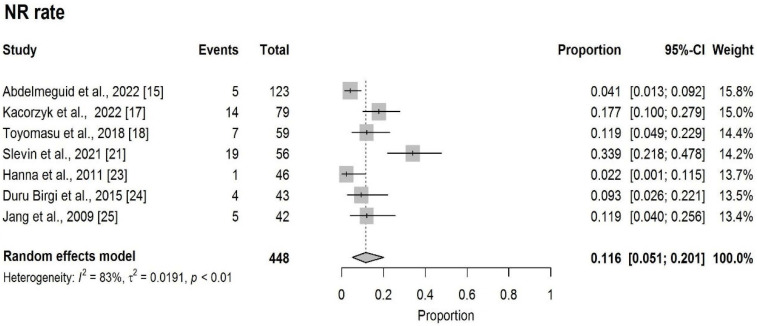
Forest plot of NR rate [15,17,18,21,23,24,25].

The pooled NR rate was 11.6% (95%CI: 5.1–20.1%) (Figure 4).

### 3.5. Distant Metastases

**Figure 5:**
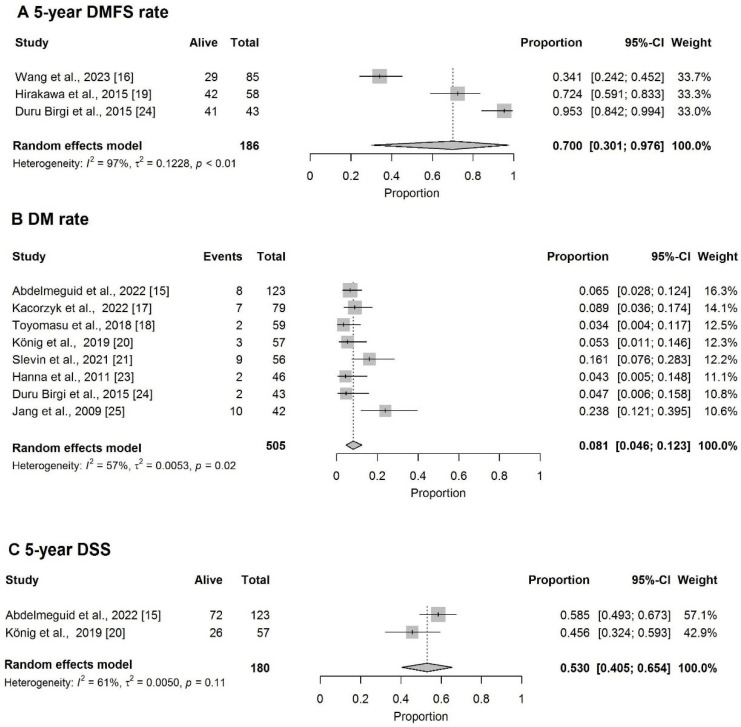
Forest plot of (**A**) 5-year DMFS rate [16,19,24] and (**B**) DM rate [15,17,18,20,21,23,24,25]. (**C**) Forest plot of 5-year DSS [15,20].

The pooled 5-year DMFS rate was 70.0% (95%CI: 30.1–97.6%) (Figure 5A) and the pooled DM rate was 8.1% (95%CI: 4.6–12.3%) (Figure 5B). The pooled 5-year DSS rate was 53% (95%CI: 40.5–65.4%) (Figure 5C).

**Figure 6:**
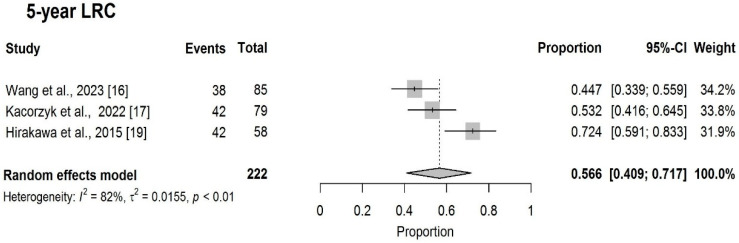
Forest plot of 5-year LRC [16,17,19].

The pooled 5-year LRC was 56.6% (95%CI: 40.9–71.7%) (Figure 6).

**Figure 7:**
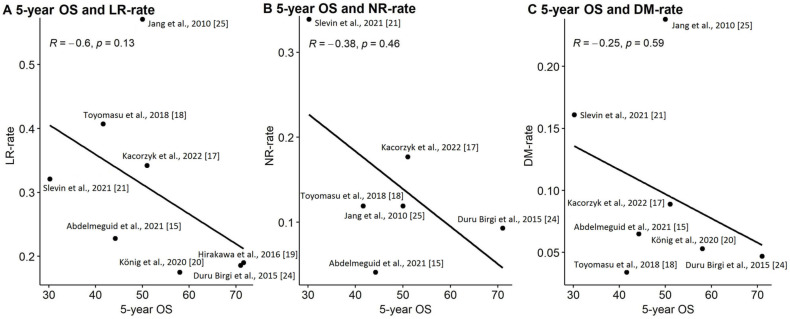
Correlation between 5-year OS and (**A**) LR [15,17,18,19,20,21,23,24,25], (**B**) NR [15,17,18,21,23,24,25], (**C**) DM rate [15,17,18,20,21,23,24,25].

There is no statistically significant correlation between 5-year OS and LR-rate (R = −0.6, *p* = 0.13), NR-rate (R = −0.38, *p* = 0.46) and DM- rate (R = −0.25, *p* = 0.59) (Figure 7).

### 3.6. Assessment of Bias Risk

The summary of the risk of bias is presented in Supplementary Materials S1. Authors’ assessments of each domain for all included studies are shown in Supplementary Materials S1, Figures S3 and S4. Overall, the included studies exhibited a low or moderate risk of bias.

## 4. Discussion

SNSCC includes about 60% of cancers arising in the sinonasal region and is the most common cancer subtype in this region [1]. Despite this, treatment recommendations are based mostly on meta-analyses and population-based studies and no randomized clinical trial has been published on this group of patients. In general, for patients with SNSCC, the 5-year OS is about 50% [1]. In our report, the 5-year OS for SNSCC patients was 50.2% and is similar to other authors, who report results between 53% and 57% [2,6,7,9,28]. Each of these meta-analyses present a wide range of results which depend on various clinical factors and treatment methods. In our study, the range of 5-year OS was between 30.4% and 72.4%. Slieker et al. showed 5-year OS rates between 25% and 82.2% for 19 studies and almost 1500 patients with SCC of the maxilla [6]. Dutta et al. also observed a wide range of treatment outcomes according to tumor localization in the sinonasal tract, with a 5-year OS of 30.2% for sphenoid sinus to 74.5% for the nasal cavity [7]. Nguyen reported a 5-year OS between 48.8% and 69.9% depending on treatment method and between 44.3% and 68.9% according to stage of disease [2].

Local recurrence (LR) is the main reason for treatment failure [4,9,29,30]. Considering the relatively low risk of nodal and distant spread of this cancer (10–15%), the achievement of local control is crucial for ultimate treatment success. Although LR and NR are often combined under the term locoregional recurrence, LR predominates, occurring in approximately 30–80% of cases depending on stage of disease and treatment modality, among other factors [29,31,32,33,34,35,36,37,38]. Our data indicated a 5-year LC rate of 57% with an LR incidence of 27.2% (range: 10.9–57.1%). Nguyen et al. reported a 5-year recurrence rate (RR) of 20.5% and a locoregional control rate of 67.1%, with an overall relapse rate (including local, regional, and distant recurrences) of 42.7% [2]. Slieker et al. reported a 5-year LR rate of 19.4% (range: 9–46.8%) for maxillary SCC [6].

According to Dooley et al., the risk of regional spread is highest for SCC followed by other histopathological subtypes like adenocarcinoma (AC), sinonasal undifferentiated carcinoma (SNUC), and adenoid cystic carcinoma (ACC), occurring in 28%, 25%, 12%, and 10% of cases, respectively [31]. Ranasinghe et al. found an overall nodal spread risk of 13.2% for SCC across all subsites, rising to 19.3% in sinus locations compared to 7.9% for the nasal cavity [39]. Our study confirmed a relatively low overall NR rate of 11.6%. Nevertheless, regional relapses are associated with significantly worse prognosis in SNSCC patients [8,40]. In fact, patients with NR often succumb to subsequent distant metastases or combined progression of local disease [8,41]. Distant metastasis is relatively uncommon for SNSCC patients, occurring in 3–20% of cases [3,9,29]. Based on our meta-analysis, the average DM risk for SNSCC patients was 8.1%. Liu et al. categorized patients with sinonasal carcinoma by their risk of distant failure, placing SNSCC alongside AC and esthesioneuroblastoma (ENB) in the low-risk group (risk of distant spread <25%), in contrast to sinonasal neuroendocrine carcinoma (SNEC), mucosal melanoma (MM), and soft tissue sarcoma (STS), which were classified as high-risk (>25%) [42]. Data from single centers indicates that patients with local or locoregional failure present significantly worse outcomes [17,43]; however, such a correlation reported from meta-analyses is less direct.

Consequently, we tried to assess how LR, NR and DM may influence OS. No correlation between these parameters was found. Similar results were obtained by Kaplan et al., who found in the studied group that DM and LR have no impact on OS; however, only 45% of patients in their study group were diagnosed with SNSCC [44]. Our observation may be due to the heterogeneity of our group, which takes into account all sites in the sinonasal region, which differ according to treatment effectiveness, most common stage of local advancement of disease or the risk of nodal spread. Primary tumor location and the probability of infiltration of nearby critical anatomical structures, like the skull base, are certainly related to the risk of treatment failure. It has also been found that the maxillary sinus is associated with a higher risk of LR than other subsites [2,45]. Also, the primary site of the tumor may influence NR with a higher risk for maxilla than ethmoid sinus infiltration. These factors influence the probability of obtaining clear resection margins (R0), detecting recurrence early or salvage effectiveness.

Recurrence diagnosed with nasal endoscopy has a higher probability of salvage surgery (SS) success than tumors diagnosed with imaging alone [46]. Salvage surgery for tumor localization in the nasal cavity or ethmoid sinuses showed better 5-OS than tumors arising in the maxillary, frontal sinuses or orbital and clivus infiltration [30,44,47].

While direct evidence linking early detection of recurrence to improved OS is lacking, for patients with SNSCC, salvage therapy is feasible in 50–70% of patients with LR and is successful in approximately 33% of cases undergoing such treatment [17,30]. The longer the time to primary treatment failure, the more effective the salvage [48]. Surgery is considered as the most effective means of salvaging but RT may also be used with good results. Kacorzyk et al. indicated 43% and 25% permanent cure after salvage surgery or RT, respectively [17]. Due to the data indicating promising results in terms of salvage for SNSCC, we also tried to analyze disease-specific survival (DSS) in this meta-analysis (Figure 5). Disease-specific survival refers to the proportion of patients who have not died from their cancer within a defined period after treatment, even despite their tumor not being cured. This parameter seems to be useful in patients with prolonged clinical disease course. The consequences of salvage may prolong clinical course of SNSCC and could be potentially seen in longer DSS. Pecorari et al.’s study indicated significantly higher DSS than OS, suggesting that patients suffering from SNSCC do not die as often because of cancer [48]. A comparison of 5-year OS and 5-year DSS for SNSCC indicates that the gap between OS and DSS is in the range of 10–40% [15,20,48,49,50]. It depends on several clinical factors including primary site of tumor, local and nodal stage of disease or treatment approach [51,52]. We found a 5-year DSS of 53% (Figure 5), which is slightly higher compared to the 50.2% 5-year OS in this group of patients Dutta et al. also reported a 5-year DSS of 52.3% in the group of 11,000 patients with SNSCC, but with no relation to OS but to relative survival (RS) [7]. Relative survival (RS) reflects the ratio between the observed survival rate and the expected survival rate in the comparable general population (matched by age, sex, or other factor). Because most survival data from patients with SNSCC comes from population-based studies where the actual cause of death is not known, RS is also commonly reported for SNSCC as an approach to DSS [48,53,54]. The RS-OS gap is in the range of 4–11% [52,53,54]. The difference between the DSS-OS gap and RS-OS gap is probably due to the fact that these two parameters rely on different assumptions: accurate determination of cause of death and adjustment of background mortality for DSS and RS, respectively. Nevertheless, higher DSS may reflect, among others, the influence of salvage on survival.

Salvage therapy for SNSCC may provide durable control in a subset of selected patients, particularly when complete resection (R0) is achievable and the optimal selection of patients for salvage seems to be pivotal for the success of the procedure, which must always maintain a balance between the chance (cure) and the risk (complications). There is not much data defining potential prognostic factors for recurrence. Kaplan et al. reported primary tumor site, histology, perineural invasion (PNI), and infiltration of carotic artery or clivus as factors significantly reducing salvage effectiveness [44]. Mattavelli et al. suggested scoring based on, among others, the type of primary treatment, histology, adjuvant treatment and postoperative histopathological results in terms of margin status and PNI [32].

## 5. Limitations

Several factors limit the strength of our conclusions. Some studies included small patient groups, while others covered long recruitment periods during which diagnostic and treatment standards evolved.

Due to the rarity of SNSCC, treatment decisions are often based on institutional experience rather than universally accepted guidelines, reducing the generalizability of findings. To mitigate these limitations, we applied strict selection criteria, although this reduced the number of included studies. Additionally, treatment outcomes depend on multiple variables, including tumor site, T and N stage, and treatment modality. A detailed analysis of these factors would enable a more individualized assessment of prognosis and treatment success.

Not all included studies reported LR, NR, and DM together with OS, which substantially reduced the number of datasets available for correlation analysis. In addition, the pooled studies were heterogeneous with respect to primary tumor site, stage distribution, nodal status, and treatment strategies, and most series were small single-institution cohorts. The included series differed substantially in primary tumor site distribution, with some studies restricted to specific subsites (e.g., maxillary sinus) and others including mixed sinonasal locations. This heterogeneity likely reflects institutional case selection and may influence pooled survival and control estimates.

The correlation analysis between LR and OS should be interpreted with caution, as the number of studies reporting both endpoints was limited, and the impact of salvage treatment on ultimate survival could not be consistently evaluated.

These factors likely reduced statistical power, may have obscured true associations between recurrence patterns and survival, and reflect the intrinsic heterogeneity of sinonasal malignancies and the challenges of conducting homogeneous meta-analyses in this rare disease setting. This heterogeneity is inherent to sinonasal malignancies and limits the interpretability of pooled estimates and correlation analyses.

Despite these limitations, this study provides a current assessment of primary treatment outcomes and highlights the potential effectiveness of salvage treatment in a selected group of patients.

## 6. Conclusions

SNSCC remains an aggressive disease with an approximately 50% 5-year OS rate in contemporary series. LR was the main reason for treatment failure, with an LR rate 27%. The pooled DM and NR rates were 8.1% and 11.6%, respectively. These findings confirm the dominant role of local failure in SNSCC and highlight the need for improved local control strategies.

## Figures and Tables

**Table 1:** Clinical characteristics of the studies included in the analysis [15,16,17,18,19,20,21,22,23,24,25,26,27].

Study ID	Country	Sample Size, Male (*n*)	Age	Primary Site n ^1^ (%)	Intervention (%) ^2^	T-Stage (%)	N-Stage (%)	Clinical Stage (%)		Median Follow-Up (Months)	Outcome (%) ^3^
			(Range;						Years of Diagnoses		
			Mean/						(Range)		
			Median)								
Abdelmeguid	USA	123 (84)	34–84; ND/60	MAX 76 (62)	iCH + SUR	T3 11	N0 71	III 9.8	1988–2017	32.6	2y OS 61.4
2022 [15]				NASAL 23 (19)	+ RT/CHRT 35	T4 89	N1 10.5	IV 90.2			3y OS 51.7
				ETHMO 16 (13)	iCH + RT/CHRT		N2 18.5				5y OS 44.2
				SPHEN 4 (3)	+/− SUR 65						2y DFS 67.9
				FRONT 2 (2)							2y DSS 67.9
				OTHER 2 (2)							3y DSS 62
											5y DSS 58.8
Wang	China	85 (63)	45–64; ND/53	MAX 40 (47.1)	SUR + RT	T4b 100	N0 65.9	ND	1999–2016	76.7	5y OS 33.9
2023 [16]				NASAL 20 (23.5)	+/− CHT 64		N + 34.1				5y DSS 38.8
				ETHMO 17 (20)	RT 36						5y DMFS 33.1
				SPHEN 4 (4.7)							5y LRC 44.6
				FRONT 4 (4.7)							
Kacorzyk	Poland	79 (50)	ND; ND/58	MAX 51 (64.5)	SUR + RT/CHRT 79	T1 6	N + 20	ND	2000–2016	34	5y OS 51
2022 [17]				NASAL 22 (28)	RT/CHRT 7	T2 29	N0 80				10y OS 44
				OTHER 6 (7.5)	ICH 14	T3 21.5					5y LC 62
						T4 43					5y NC 75
											5y LRC 53
Toyomasu	Japan	59 (45)	35–92; ND/60	MAX 29 (49)	PT 64	T1 3	N0 83	I 3	2001–2012	30	3y OS 56.2
2018 [18]				ETHMO 18 (13)	CIT 36	T2 3	N1 3	II 3			5y OS 41.6
				NASAL 6 (10)		T3 14	N2 3	III 14			3y LC 54
				FRONT 4 (7)		T4 70		IV 70			5y LC 50.4
				SPHEN 2 (3)							
Hirakawa	Japan	58 (51)	35–77; ND/60	MAX 53 (91.4)	SUR 17	T2 5	N0 79	II 5	2000–2009	40	5y OS 71.6
2015 [19]				ETHMO 5 (8.6)	iCH + SUR 63	T3 24	N1 7	III 19			5y DMFS 72.8
					iCH + SUR + RT 10	T4 71	N2 12	IV 76			5y LRC 73
					SUR + RT 9		N3 2				5y DFS 67.9
König	Norway	57 (ND)	ND; ND	SINONASAL 57 (100)	RT + SUR 8.8	ND	ND	ND	1988–2017	42	2y OS 81
2019 [20]					CHRT + SUR 1.8						5y OS 58
					SUR + RT 31.6						10y OS 49
					SUR + CHRT 8.8						2y DSS 81
					SUR 8.8						5y DSS 65
					RT 21						10y DSS 54
					CHRT 19						
Slevin	UK	56 (38)	39–85; ND/60	MAX 39 (70)	SUR 5	T2 2	N + 36	ND	2012–2017	45.6	3y OS 63.2
2021 [21]				NASAL 12 (21)	SUR + CHRT 5	T3 4					5y OS 30.2
				ETHMO/FRONT	SUR + RT 38	T4 94					
				5 (9)	iCH + SUR 5						
					iCH + RT 12						
					RT 20						
					CHRT 10						
					iCH + CHRT 4						
Anjum	Pakistan	46 (ND)	ND; ND	SINONASAL 46 (100)	SUR 28.3	ND	N + 43	II 13	2001–2015	34	2y OS 48
2019 [22]								III 17			3y OS 29
								IV 70			
Hanna	USA	46 (ND)	ND; ND/59	MAX 31 (67)	iCH + SUR + RT 32.6	T3 19.6	N + 26	III 20	ND	ND	2y OS 67
2011 [23]				ETHMO 9 (20)	iCH + SUR + CHRT 11	T4 80.4		IV 80			
				NASAL 4 (9)	iCH + RT 30.4						
				SPHEN 2 (4)	iCH + CHRT 17.4						
					iCH + CHRT + SUR 4.3						
					iCH 4.3						
Duru Birgi	UK	43 (25)	ND; ND	MAX 20 (47)	iCH + RT 7	T1 14	N0 88	I 14	2007–2012	32	2y OS 80
2015 [24]				ETHMO 2 (2)	CHRT 7	T2 14	N1 9	II 14			5y OS 71
				NASAL 21 (51)	SUR + RT 58	T3 5	N2 3	III 5			2y LC 81
					RT 28	T4 67		IV 67			5y LC 76
											2y DMFS 95
											5y DMFS 95
											2y DSS 84
											5y DSS 74
Jang	Republic of Korea	42 (36)	ND; ND	NASAL 12 (28.5)	iCH + RT 64	T3 23.8	N0 100	III 23.8	1990–2007	38	5y OS 34 MAX
2009 [25]				MAX 30 (71.5)	iCH + CHRT 2.4	T4 76.2		IV 76.2		for surviving	5y LC 29 MAX
					CHRT 12					patients	5y OS 50 NASAL
					RT 19						5y LC 52 NASAL
											5y DMFS 78 MAX
											5y DMFS 47
											NASAL
Saito	Japan	37 (29)	32–81; ND/65	NASAL 19	iCH +PT 48.64	T3 10.8	N0 94.6	ND	2003–2020	53	4y OS 58
2023 [26]				MAX 40.5	PT 24.32	T4 89.2	N+ 6.4				4y PFS 43
				ETHMO 35	iCH + SUR + PT 5.4						4y LC 58
				SPHEN 51	SUR + PT 21.62						
Abe	Japan	18 (12)	46–83; ND/67	MAX 18 (100)	CHRT 100	T3 22	N + 100	III 17	2008–2019	17	2y OS 46
2020 [27]						T4 78		IV 83			2y LC 34

Abbreviations: 1. NASAL—nasal cavity; SINONASAL—paranasal sinuses, sinonasal region; ETHMO—ethmoid sinus; MAX—maxillary sinus; SPHEN—sphenoid sinus; FRONT—frontal sinus; OTHER—other region of head and neck; 2. CHRT—chemoradiotherapy; CH—chemotherapy; iCH—induction chemotherapy; PT—particle beam radiation therapy/proton therapy; CIT—carbon ion therapy; RT—radiotherapy; SUR—surgery; ND—no data; SCC—squamous cell carcinoma; 3. DFS—disease-free survival; DMFS—distant metastasis-free survival; DSS—disease-specific survival; LC—local control; LRC—locoregional control; NC—nodal control; OS—overall survival; T—tumor; N—nodes; y—year.

## Data Availability

All data supporting the findings of this study are available within the paper and its Supplementary Information.

## Supplementary Materials

The following supporting information can be downloaded at https://www.mdpi.com/article/10.3390/cancers18060948/s1: Table S1A. Search terms; Table S1B. Limits used for search strategy; Table S2. Search strategy for PubMed; Table S3A. Search strategy for Scopus without quotation marks; Table S3B. Search strategy for Scopus with quotation marks; Table S4A. Search strategy for Web of Science without quotation marks; Table S4B. Search strategy for Web of Science with quotation marks; Figure S1. Forest plot of 2-year OS rate; Figure S2. Forest plot of 3-year OS rate; Figure S3. Risk of bias graph showing review author’s judgment about each risk of bias item presented as percentages across all included studies; Figure S4. Risk of bias summary showing review author’s judgment about each risk of bias item for each included study.
