# Supplementary material for: Treatment Failure and Overall Survival in Patients with Sinonasal Squamous Cell Carcinoma (SNSCC): A Systematic Review and Meta-Analysis
Source: Cancers (Basel). 2026 Mar 13;18(6):948. doi: 10.3390/cancers18060948 (PMC13024341; doi:10.3390/cancers18060948)
Supplement: Supplementary file 1 [file cancers-18-00948-s001.zip › cancers-4151071-supplementary for xml.pdf]

Supplementary Table S1A. Search terms

No	Search terms
1	**Cancer of maxilla and sinonasal region**
2	**Sinonasal malignancy**
3	**Neoplasms of the maxillo-ethmoid massif**
4	**Maxillary sinus squamous cell carcinoma**
5	**Cancer of nasal cavity and paranasal sinuses**
6	**Sinonasal cancer**
7	**Paranasal sinuses malignancies**
8	**Nasal and paranasal sinus carcinoma**
9	**Malignant tumors of the nasal cavity, ethmoid and sphenoid sinuses**
10	**Malignant neoplasms of the sinonasal tract**
11	**paranasal sinus neoplasms**
12	**maxillary sinus neoplasms**
13	**ethmoid sinus neoplasms**
14	**sphenoid sinus neoplasms**
15	**frontal sinus neoplasms**
16	**surgery**
17	**radiotherapy**
18	**radical treatment**
19	**definitive treatment**

Supplementary Table S1B. Limits used for search strategy

No	Search limits
1	articles written in English(MEDLINE/PubMed, Scopus & WOS)
2	full text articles (MEDLINE/PubMed)
3	articles (Scopus & WOS)
4	final publication stage (Scopus)
5	publications after year 2000 (MEDLINE/PubMed, Scopus & WOS)

Supplementary Table S2. Search strategy for PubMed

Search	Query	Hits February 28, 2025
#1	Search: **Cancer of maxilla and sinonasal region**<br>„Cancer of maxilla"[Title/Abstract] AND „sinonasal region"[Title/Abstract]	0
#2	Search: **Sinonasal malignancy**<br>"sinonasal malignancy"[Title/Abstract]	279
#3	Search: **Neoplasms of the maxillo-ethmoid massif**<br>Neoplasms of the maxillo-ethmoid massif[Title/Abstract]	0
#4	Search: **Maxillary sinus squamous cell carcinoma**<br>"maxillary sinus squamous cell carcinoma"[Title/Abstract]	75
#5	Search: **Cancer of nasal cavity and paranasal sinuses**<br>("cancer s"[All Fields] OR "cancerated"[All Fields] OR "canceration"[All Fields] OR "cancerization"[All Fields] OR "cancerized"[All Fields] OR "cancerous"[All Fields] OR "neoplasms"[MeSH Terms] OR "neoplasms"[All Fields] OR "cancer"[All Fields] OR "cancers"[All Fields]) AND "of nasal cavity"[Title/Abstract] AND "paranasal sinuses"[Title/Abstract]	2
#6	Search: **Sinonasal cancer**<br>"sinonasal cancer"[Title/Abstract]	404

#7	Search: **Paranasal sinuses malignancies**<br>"paranasal sinuses malignancies"[Title/Abstract]	2
#8	Search: **Nasal and paranasal sinus carcinoma**<br>"Nasal"[Title/Abstract] AND "paranasal sinus carcinoma"[Title/Abstract]	18
#9	Search: **Malignant tumors of the nasal cavity, ethmoid and sphenoid sinuses**<br>Malignant tumors of the nasal cavity, ethmoid[Title/Abstract] AND sphenoid sinuses[Title/Abstract]	0
#10	Search: **Malignant neoplasms of the sinonasal tract**<br>("neoplasms"[MeSH Terms] OR "neoplasms"[All Fields] OR ("malignant"[All Fields] AND "neoplasms"[All Fields]) OR "malignant neoplasms"[All Fields]) AND "of the sinonasal tract"[Title/Abstract]	1
#11	Search: **paranasal sinus neoplasms**<br>"paranasal sinus neoplasms"[Title/Abstract]	305
#12	Search: **maxillary sinus neoplasms**<br>"maxillary sinus neoplasms"[Title/Abstract]	187
#13	Search: **ethmoid sinus neoplasms**<br>"ethmoid sinus neoplasms"[Title/Abstract]	40
#14	Search: **sphenoid sinus neoplasms**<br>"sphenoid sinus neoplasms"[Title/Abstract]	10
#15	Search: **frontal sinus neoplasms**<br>"frontal sinus neoplasms"[Title/Abstract]	56
#16	Search: **surgery**<br>"surgery"[Title/Abstract]	1,627,180
#17	Search: **radiotherapy**<br>"radiotherapy"[Title/Abstract]	239,190
#18	Search: **radical treatment**<br>"radical treatment"[Title/Abstract]	3,719
#19	Search: **definitive treatment**<br>"definitive treatment"[Title/Abstract]	11,638
#20	OR/#2, #4-#8, #10-#15	1,316
#21	#16 OR #17	1,806,842
#22	#18 OR #19	15,323
#23	#20 AND #21	529
#24	#23 AND #22	11
#25	limit #16 to english	11
#26	limit #17 to full text	11
#27	limit #19 to publications after year 2000	11
**#27**	**Full Query**	11
	Search: **((((((((((((Sinonasal malignancy[Title/Abstract]) OR (Maxillary sinus squamous cell carcinoma[Title/Abstract])) OR (Cancer of nasal cavity[Title/Abstract] AND paranasal sinuses[Title/Abstract])) OR (Sinonasal cancer[Title/Abstract])) OR (Paranasal sinuses malignancies[Title/Abstract])) OR (Nasal[Title/Abstract] AND paranasal sinus carcinoma[Title/Abstract])) OR (Malignant neoplasms of the sinonasal tract[Title/Abstract])) OR (Maxillary Sinus Neoplasms[Title/Abstract])) OR (Paranasal Sinus Neoplasms[Title/Abstract])) OR (sphenoid sinus neoplasms[Title/Abstract])) OR (frontal sinus neoplasms[Title/Abstract])) OR (Ethmoid Sinus Neoplasms[Title/Abstract])) AND ((surgery[Title/Abstract]) OR (radiotherapy[Title/Abstract]))) AND ((radical treatment[Title/Abstract]) OR (definitive treatment[Title/Abstract])) Filters: Full text, English, from 2000 - 2022**<br>(("sinonasal malignancy"[Title/Abstract] OR "maxillary sinus squamous cell carcinoma"[Title/Abstract] OR (("cancer s"[All Fields] OR "cancerated"[All Fields] OR "canceration"[All Fields] OR "cancerization"[All Fields] OR "cancerized"[All Fields] OR "cancerous"[All Fields] OR "Neoplasms"[MeSH Terms] OR "Neoplasms"[All Fields] OR "cancer"[All Fields] OR "cancers"[All Fields]) AND "of nasal cavity"[Title/Abstract] AND "paranasal sinuses"[Title/Abstract]) OR "sinonasal cancer"[Title/Abstract] OR "paranasal sinuses malignancies"[Title/Abstract] OR ("Nasal"[Title/Abstract] AND "paranasal sinus carcinoma"[Title/Abstract]) OR (("Neoplasms"[MeSH Terms] OR "Neoplasms"[All Fields] OR ("malignant"[All Fields] AND "Neoplasms"[All Fields]) OR "malignant neoplasms"[All Fields]) AND "of the sinonasal tract"[Title/Abstract]) OR "maxillary sinus neoplasms"[Title/Abstract] OR "paranasal sinus neoplasms"[Title/Abstract] OR "sphenoid sinus	

	neoplasms"[Title/Abstract] OR "frontal sinus neoplasms"[Title/Abstract] OR "ethmoid sinus neoplasms"[Title/Abstract]) AND ("surgery"[Title/Abstract] OR "radiotherapy"[Title/Abstract]) AND ("radical treatment"[Title/Abstract] OR "definitive treatment"[Title/Abstract])) AND ((fft[Filter]) AND (english[Filter]) AND (2000:2025[pdat]))	

Supplementary Table S3A. Search strategy for Scopus without quotation marks

Search	Query	Hits February 28, 2025
#1	TITLE-ABS-KEY (cancer AND of AND maxilla AND sinonasal AND region)	59
#2	TITLE-ABS-KEY (sinonasal AND malignancy)	1,566
#3	TITLE-ABS-KEY (neoplasms AND of AND the AND maxillo-ethmoid AND massif)	0
#4	TITLE-ABS-KEY (maxillary AND sinus AND squamous AND cell AND carcinoma)	1,617
#5	TITLE-ABS-KEY (cancer AND of AND nasal AND cavity AND paranasal AND sinuses)	2,048
#6	TITLE-ABS-KEY (sinonasal AND cancer)	3,806
#7	TITLE-ABS-KEY (paranasal AND sinuses AND malignancies)	1,907
#8	TITLE-ABS-KEY (nasal AND paranasal AND sinus AND carcinoma)	2,008
#9	TITLE-ABS-KEY (malignant AND tumors AND of AND the AND nasal AND cavity, AND ethmoid AND sphenoid AND sinuses)	90
#10	TITLE-ABS-KEY (malignant AND neoplasms AND of AND the AND sinonasal AND tract)	273
#11	TITLE-ABS-KEY (paranasal AND sinus AND neoplasms)	11,338
#12	TITLE-ABS-KEY (maxillary AND sinus AND neoplasms)	5,503
#13	TITLE-ABS-KEY (ethmoid AND sinus AND neoplasms)	2,700
#14	TITLE-ABS-KEY (sphenoid AND sinus AND neoplasms)	3,101
#15	TITLE-ABS-KEY (frontal AND sinus AND neoplasms)	2,204
#16	TITLE-ABS-KEY (surgery)	2,994,321
#17	TITLE-ABS-KEY (radiotherapy)	530,589
#18	TITLE-ABS-KEY (radical AND treatment)	175,867
#19	TITLE-ABS-KEY (definitive AND treatment)	71,521
#20	OR/#1, #2 #4-#15	20,531
#21	#16 OR #17	3,357,064
#22	#18 OR #19	244,508
#23	#20 AND #21	10,329
#24	#23 AND #22	715
#25	limit #17 to english	596
#26	limit #18 to article	506
#27	limit #19 to final publication stage	503
#28	limit #20 to publications after year 2000	419
**#23**	**Full Query**	**419**
	(((TITLE-ABS-KEY (cancer AND of AND maxilla AND sinonasal AND region)) OR (TITLE-ABS-KEY (sinonasal AND malignancy)) OR (TITLE-ABS-KEY (neoplasms AND of AND the AND maxillo-ethmoid AND massif)) OR (TITLE-ABS-KEY (maxillary AND sinus AND squamous AND cell AND carcinoma)) OR (TITLE-ABS-KEY (cancer AND of AND nasal AND cavity AND paranasal AND sinuses)) OR (TITLE-ABS-KEY (sinonasal AND cancer)) OR (TITLE-ABS-KEY (paranasal AND sinuses AND malignancies)) OR (TITLE-ABS-KEY (nasal AND paranasal AND sinus AND carcinoma)) OR (TITLE-ABS-KEY (malignant AND tumors AND of AND the AND nasal AND cavity, AND ethmoid AND sphenoid AND sinuses)) OR (TITLE-ABS-KEY (malignant AND neoplasms AND of AND the AND sinonasal AND tract)) OR (TITLE-ABS-KEY (paranasal AND sinus AND neoplasms)) OR (TITLE-ABS-KEY (maxillary AND sinus AND neoplasms)) OR (TITLE-ABS-KEY (ethmoid AND sinus AND neoplasms)) OR (TITLE-ABS-KEY (sphenoid AND sinus AND neoplasms)) OR (TITLE-ABS-KEY (frontal AND sinus AND neoplasms))) AND ((TITLE-ABS-KEY (surgery)) OR (TITLE-ABS-KEY (radiotherapy)))) AND ((TITLE-ABS-KEY (radical AND treatment)) OR (TITLE-ABS-KEY (definitive AND treatment))) AND PUBYEAR > 1999 AND PUBYEAR < 2026 AND (LIMIT-TO (LANGUAGE,"English")) AND (LIMIT-TO (DOCTYPE,"ar")) AND (LIMIT-TO (PUBSTAGE,"final"))	

Supplementary Table S3B. Search strategy for Scopus with quotation marks

Search	Query	Hits February 28, 2025
#1	TITLE-ABS-KEY („Cancer of maxilla and sinonasal region”)	0
#2	TITLE-ABS-KEY („Sinonasal malignancy”)	622
#3	TITLE-ABS-KEY („Neoplasms of the maxillo-ethmoid massif”)	0
#4	TITLE-ABS-KEY („Maxillary sinus squamous cell carcinoma”)	93
#5	TITLE-ABS-KEY („Cancer of nasal cavity and paranasal sinuses")	5
#6	TITLE-ABS-KEY („Sinonasal cancer”)	598
#7	TITLE-ABS-KEY („Paranasal sinuses malignancies”)	80
#8	TITLE-ABS-KEY („Nasal and paranasal sinus carcinoma”)	15
#9	TITLE-ABS-KEY („Malignant tumors of the nasal cavity, ethmoid and sphenoid sinuses”)	0
#10	TITLE-ABS-KEY („Malignant neoplasms of the sinonasal tract”)	12
#11	TITLE-ABS-KEY („paranasal sinus neoplasms”)	7,420
#12	TITLE-ABS-KEY („maxillary sinus neoplasms”)	1,852
#13	TITLE-ABS-KEY („ethmoid sinus neoplasms”)	33
#14	TITLE-ABS-KEY („sphenoid sinus neoplasms”)	12
#15	TITLE-ABS-KEY („frontal sinus neoplasms”)	65
#16	TITLE-ABS-KEY („surgery”)	2,994,321
#17	TITLE-ABS-KEY („radiotherapy”)	530,589
#18	TITLE-ABS-KEY („radical treatment”)	5,285
#19	TITLE-ABS-KEY („definitive treatment”)	13,572
#20	OR/#1-15	9,655
#21	#16 OR #17	3,357,064
#22	#18 OR #19	18,815
#23	#20 AND #21	4,363
#24	#22 AND #23	29
#25	limit #24 to english	27
#26	limit #25 to article	22
#27	limit #26 to final publication stage	22
#28	limit #27 to publications after year 2000	17
**#29**	**Full Query**	**17**
	((TITLE-ABS-KEY ("radical treatment")) OR (TITLE-ABS-KEY ("definitive treatment"))) AND (((TITLE-ABS-KEY ("frontal sinus neoplasms")) OR (TITLE-ABS-KEY ("sphenoid sinus neoplasms")) OR (TITLE-ABS-KEY ("ethmoid sinus neoplasms")) OR (TITLE-ABS-KEY ("maxillary sinus neoplasms")) OR (TITLE-ABS-KEY ("paranasal sinus neoplasms")) OR (TITLE-ABS-KEY ("Malignant neoplasms of the sinonasal tract")) OR (TITLE-ABS-KEY ("Malignant tumors of the nasal cavity, ethmoid and sphenoid sinuses")) OR (TITLE-ABS-KEY ("Nasal and paranasal sinus carcinoma")) OR (TITLE-ABS-KEY ("Paranasal sinuses malignancies")) OR (TITLE-ABS-KEY ("Sinonasal cancer")) OR (TITLE-ABS-KEY ("Cancer of nasal cavity and paranasal sinuses")) OR (TITLE-ABS-KEY ("Maxillary sinus squamous cell carcinoma")) OR (TITLE-ABS-KEY ("Neoplasms of the maxillo-ethmoid massif")) OR (TITLE-ABS-KEY ("Sinonasal malignancy")) OR (TITLE-ABS-KEY ("Cancer of maxilla and sinonasal region"))) AND ((TITLE-ABS-KEY ("surgery")) OR (TITLE-ABS-KEY ("radiotherapy")))) AND PUBYEAR > 1999 AND PUBYEAR < 2026 AND (LIMIT-TO (PUBSTAGE,"final")) AND (LIMIT-TO (DOCTYPE,"ar")) AND (LIMIT-TO (LANGUAGE,"English"))	

Supplementary Table S4A. Search strategy for Web of Science without quotation marks

Search	Query	Hits February 28, 2025
#1	(TI=(Cancer of maxilla and sinonasal region)) OR AB=(Cancer of maxilla and sinonasal region)	1
#2	(TI=(Sinonasal malignancy)) OR AB=(Sinonasal malignancy)	1,164
#3	(TI=(Neoplasms of the maxillo-ethmoid massif)) OR AB=(Neoplasms of the maxillo-ethmoid massif)	0
#4	(TI=(Maxillary sinus squamous cell carcinoma)) OR AB=(Maxillary sinus squamous cell carcinoma)	456
#5	(TI=(Cancer of nasal cavity and paranasal sinuses)) OR AB=(Cancer of nasal cavity and paranasal sinuses)	269
#6	(TI=(Sinonasal cancer)) OR AB=(Sinonasal cancer)	1,028
#7	(TI=(Paranasal sinuses malignancies)) OR AB=(Paranasal sinuses malignancies)	556
#8	(TI=(Nasal and paranasal sinus carcinoma)) OR AB=(Nasal and paranasal sinus carcinoma)	507
#9	(TI=(Malignant tumors of the nasal cavity, ethmoid and sphenoid sinuses)) OR AB=(Malignant tumors of the nasal cavity, ethmoid and sphenoid sinuses)	30
#10	(TI=(Malignant neoplasms of the sinonasal tract)) OR AB=(Malignant neoplasms of the sinonasal tract)	87
#11	(TI=(Paranasal Sinus Neoplasms)) OR AB=(Paranasal Sinus Neoplasms)	535
#12	(TI=(Maxillary Sinus Neoplasms)) OR AB=(Maxillary Sinus Neoplasms)	343
#13	(TI=(Ethmoid Sinus Neoplasms)) OR AB=(Ethmoid Sinus Neoplasms)	162
#14	(TI=(sphenoid sinus neoplasms)) OR AB=(sphenoid sinus neoplasms)	147
#15	(TI=(frontal sinus neoplasms)) OR AB=(frontal sinus neoplasms)	125
#16	(TI=(surgery)) OR AB=(surgery)	1,465,513
#17	(TI=(radiotherapy)) OR AB=(radiotherapy)	243,338
#18	(TI=(radical treatment)) OR AB=(radical treatment)	94,699
#19	(TI=(definitive treatment)) OR AB=(definitive treatment)	47,010
#20	OR/#1, #2 #4-#15	3,950
#21	#16 OR #17	1,657,971
#22	#18 OR #19	139,955
#23	#20 AND #21	1,541
#24	#23 AND #22	157
#25	limit #24 to english	150
#26	limit #25 to article	126
#27	limit #26 to publications after year 2000	114
**#27**	**Full Query**	**114**
	((TI=(Cancer of maxilla and sinonasal region)) OR AB=(Cancer of maxilla and sinonasal region) OR (TI=(Sinonasal malignancy)) OR AB=(Sinonasal malignancy) OR (TI=(Maxillary sinus squamous cell carcinoma)) OR AB=(Maxillary sinus squamous cell carcinoma) OR (TI=(Cancer of nasal cavity and paranasal sinuses)) OR AB=(Cancer of nasal cavity and paranasal sinuses) OR (TI=(Sinonasal cancer)) OR AB=(Sinonasal cancer) OR (TI=(Paranasal sinuses malignancies)) OR AB=(Paranasal sinuses malignancies) OR (TI=(Nasal and paranasal sinus carcinoma)) OR AB=(Nasal and paranasal sinus carcinoma) OR (TI=(Malignant tumors of the nasal cavity, ethmoid and sphenoid sinuses)) OR AB=(Malignant tumors of the nasal cavity, ethmoid and sphenoid sinuses) OR (TI=(Malignant neoplasms of the sinonasal tract)) OR AB=(Malignant neoplasms of the sinonasal tract) OR (TI=(Paranasal Sinus Neoplasms)) OR AB=(Paranasal Sinus Neoplasms) OR (TI=(Maxillary Sinus Neoplasms)) OR AB=(Maxillary Sinus Neoplasms) OR (TI=(Ethmoid Sinus Neoplasms)) OR AB=(Ethmoid Sinus Neoplasms) OR (TI=(sphenoid sinus neoplasms)) OR AB=(sphenoid sinus neoplasms) OR (TI=(frontal sinus neoplasms)) OR AB=(frontal sinus neoplasms)) AND ((TI=(surgery)) OR AB=(surgery) OR (TI=(radiotherapy)) OR AB=(radiotherapy)) AND ((TI=(radical treatment)) OR AB=(radical treatment) OR (TI=(definitive treatment)) OR AB=(definitive treatment)) AND LA==("ENGLISH") AND DT==("ARTICLE") AND PY=(2000-2025)	

Supplementary Table S4B. Search strategy for Web of Science with quotation marks

Search	Query	February 28, 2025
#1	TS=("Cancer of maxilla and sinonasal region")	0
#2	TS=("Sinonasal malignancy")	228
#3	TS=("Neoplasms of the maxillo-ethmoid massif")	0
#4	TS=("Maxillary sinus squamous cell carcinoma")	68
#5	TS=("Cancer of nasal cavity and paranasal sinuses")	2
#6	TS=("Sinonasal cancer ")	572
#7	TS=("Paranasal sinuses malignancies")	4
#8	TS=("Nasal and paranasal sinus carcinoma")	3
#9	TS=("Malignant tumors of the nasal cavity, ethmoid and sphenoid sinuses")	0
#10	TS=("Malignant neoplasms of the sinonasal tract")	6
#11	TS=("paranasal sinus neoplasms")	140
#12	TS=("maxillary sinus neoplasms")	48
#13	(TS=("ethmoid sinus neoplasms")	0
#14	TS=("sphenoid sinus neoplasms")	1
#15	TS=("frontal sinus neoplasms")	2
#16	TS=("surgery")	1,726,982
#17	TS=("radiotherapy")	326,178
#18	TS=("radical treatment ")	3,356
#19	TS=("definitive treatment")	10,648
#20	OR/#1- #15	1,068
#21	#16 OR #17	1,975,346
#22	#18 OR #19	13,967
#23	#20 AND #21	517
#24	#23 AND #22	14
#25	limit #24 to english	14
#26	limit #25 to article	14
#27	limit #26 to publications after year 2000	14
**#27**	**Full Query**	**14**
	(TS=("Cancer of maxilla and sinonasal region") OR TS=("Sinonasal malignancy") OR TS=("Neoplasms of the maxillo-ethmoid massif") OR TS=(Maxillary sinus squamous cell carcinoma) OR TS=("Cancer of nasal cavity and paranasal sinuses") OR TS=("Sinonasal cancer ") OR TS=("Paranasal sinuses malignancies") OR TS=("Nasal and paranasal sinus carcinoma") OR TS=("Malignant tumors of the nasal cavity, ethmoid and sphenoid sinuses") OR TS=("Malignant neoplasms of the sinonasal tract ") OR TS=("paranasal sinus neoplasms") OR TS=("maxillary sinus neoplasms") OR TS=("ethmoid sinus neoplasms") OR TS=(" sphenoid sinus neoplasms") OR TS=("frontal sinus neoplasms")) AND (TS=("radical treatment ") OR TS=("definitive treatment")) AND (TS=("surgery") OR TS=("radiotherapy")) AND LA==("ENGLISH") AND DT==("ARTICLE") AND PY=(2000-2025)	

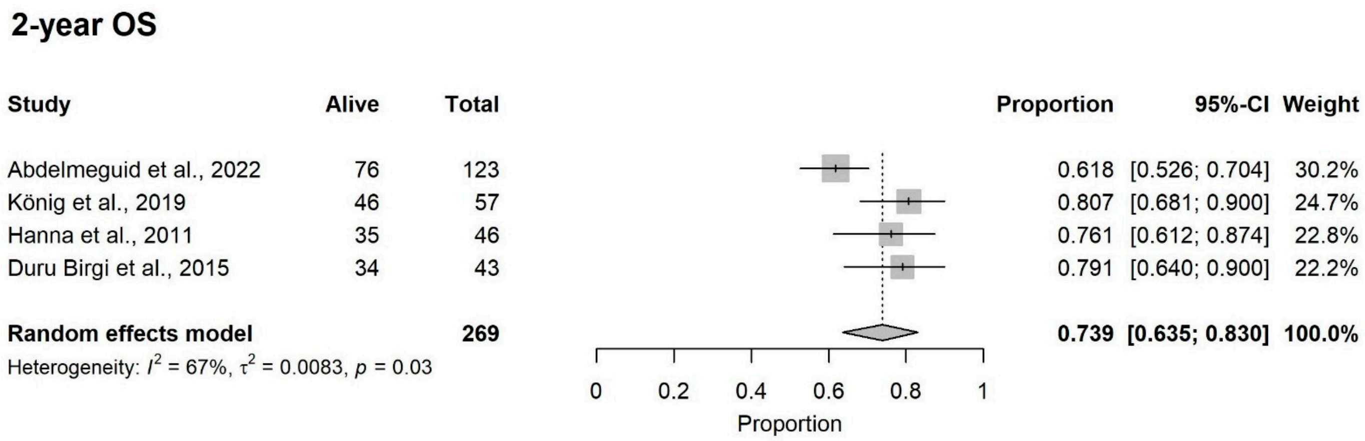

Figure S1. Forest plot of 2-year OS rate [15,20,23,24].

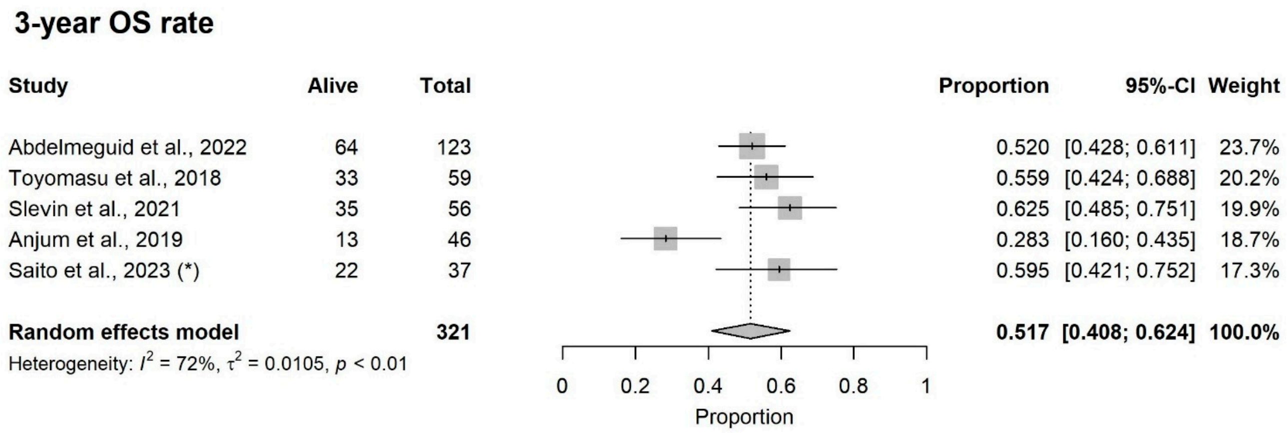

Figure S2. Forest plot of 3-year OS rate [15,18,21,26].

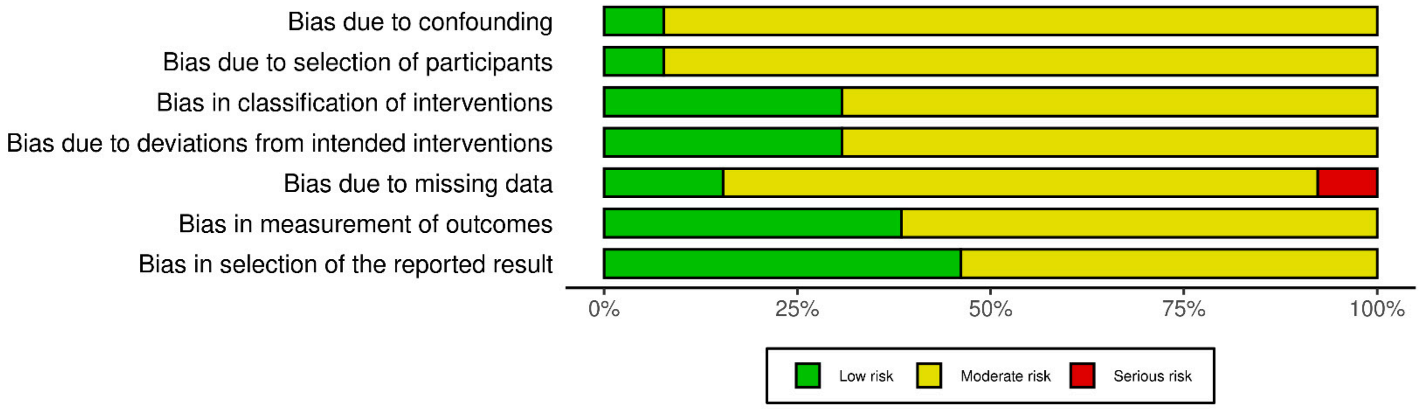

Figure S3. Risk of bias graph showing review author's judgment about each risk of bias item presented as percentages across all included studies

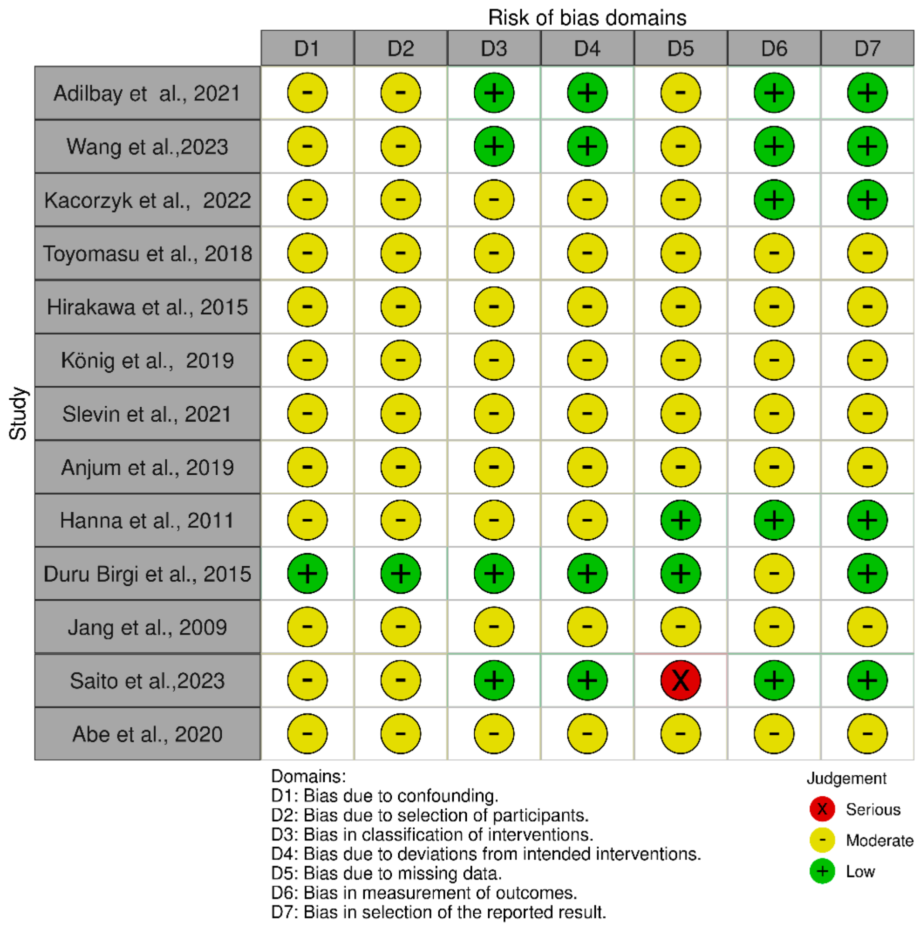

Figure S4. Risk of bias summary showing review author's judgment about each risk of bias item for each included study [15-27].
